# Synergy Makes Direct Perception Inefficient

**DOI:** 10.3390/e26080708

**Published:** 2024-08-21

**Authors:** Miguel de Llanza Varona, Manolo Martínez

**Affiliations:** 1 School of Engineering and Informatics, University of Sussex, Brighton BN1 9RH, UK; 2 Philosophy Department, Universitat de Barcelona, 08001 Barcelona, Spain; manolomartinez@ub.edu

**Keywords:** synergy, affordances, direct perception, ecological information

## Abstract

A typical claim in anti-representationalist approaches to cognition such as ecological psychology or radical embodied cognitive science is that ecological information is sufficient for guiding behavior. According to this view, affordances are immediately perceptually available to the agent (in the so-called “ambient energy array”), so sensory data does not require much further inner processing. As a consequence, mental representations are explanatorily idle: perception is immediate and direct. Here we offer one way to formalize this direct-perception claim and identify some important limits to it. We argue that the claim should be read as saying that successful behavior just implies picking out affordance-related information from the ambient energy array. By relying on the Partial Information Decomposition framework, and more concretely on its development of the notion of synergy, we show that in multimodal perception, where various energy arrays carry affordance-related information, the “just pick out affordance-related information” approach is very inefficient, as it is bound to miss all synergistic components. Efficient multimodal information combination requires transmitting sensory-specific (and not affordance-specific) information to wherever it is that the various information streams are combined. The upshot is that some amount of computation is necessary for efficient affordance reconstruction.

## 1. Introduction

Cognition is often taken to be (among other things, but centrally) involved in the generation of “adaptive behavior” ([1], ([2] p. 359)]), which is sensitive to “the structure of the environment and the goals of the [cognitive agent]” ([3], p. 3). One natural way to think of cognition, then, is as the transformation and combination of information relevant to the production of behavior (some of it incoming from the environment, some of it encoding agent goals, etc.) into an actual moment-by-moment behavioral plan.

The most popular approach to the investigation of this process is what [4] calls *mainstream representationalism* [5,6,7]: the view that this transmission and combination of information depends on computations over representations. What exactly representations are is a matter of much debate; for our current purposes, we can simply think of them as signals that carry information about, among other things, the agent’s current environment, or their current goals, to downstream areas where these streams of information are combined and transformed in ways increasingly relevant to the production of behavior.

While representationalism is both popular and scientifically successful [8], it is not the only game in town. Alternatives to representationalist cognitive science include *radical embodied* [9,10] cognitive science. This approach is part of a package of views in cognitive science that is steadily gaining in influence: so-called *4E* approaches to cognition [11] downplay the importance of internal computation, and highlight the fact that, sometimes at least, behavior-relevant information can be simply picked up from the environment with very little “post-processing”. This shift of focus has allowed embodied cognitive scientists, for example, to redescribe interceptive actions, such as a baseball outfielder catching a ball [10,12]: instead of the outfielder’s brain solving the physics problem of predicting the position and time at which the ball will impact the ground from some estimated initial conditions, the outfielder can simply “align themselves with the path of the ball and run so as to make the ball appear to move with constant velocity” ([10], p. 5). This is less computationally intensive, and potentially more ecologically plausible, than the kind of physics-based calculations that classical cognitive science would traditionally gravitate towards.

Embodied cognitive science, therefore, stresses the role that agent–environment dynamics play in cognition. We talk of “stressing the role” rather than “substituting representations with” advisedly: we don’t think that representationalism and these alternative approaches are in conflict—perhaps *contra* their proponents, and the overall tenor of the debate surrounding them. Rather we believe, with [13], that they should be thought of as complementary, largely compatible tools in the cognitive-scientific toolbox.

Under this light, one important task for theorists of cognitive science consists in charting the range of applicability of these different approaches: that they are all useful certainly need not mean that they all be everywhere and universally useful. It might very well be, for example, that representation-based analyses happen not to be illuminating in the description and explanation of some particular cognitive process (e.g., perhaps sudden “Aha!” moments of mathematical insight, as described in [14]), and it might equally well be that there are limits to the explanatory usefulness of non-representational strategies.

In this paper, in particular, we discuss, from this vantage point, one of the main themes in radical embodied cognitive science and ecological psychology [15,16]: the claims that, first, the contents of perception are determined by a set of regularities present in the environment, called “affordances” [17]; and, second, that information about affordances can be *directly perceived* by the agent, without the need for any inner processing or computation [15,18,19]. Here we will show that there are some limits to this putatively direct, non-computational, non-representational information pickup.

## 2. The Direct Perception of Affordances

In keeping with the notion, discussed above, that cognition is intimately linked to the generation of adaptive behavior, radical embodied cognitive scientists and ecological psychologists think of perception as being essentially for action: agents explore their environment so that, through action, they can modify it. Specifically, agents actively engage with their environment through the perception of affordances: possibilities for action *afforded by* the environment, such as climbability (that affords climbing), drinkability (that affords drinking), etc.

How do we perceive affordances? There is a “set of structures and regularities in the environment that allow an animal to engage with [them]” ([19], p. 5232). These structures and regularities are what ecological psychologists call *ecological information*. Ecological information inheres on an *ambient energy array*: highly structured patterns of, e.g., ambient light, or of sound waves, that carry information about present affordances [17]. What we may call, in turn, the *direct perception hypothesis* [20] is the claim that perceivers can directly pick up this ecological information in the environment without the need to compute over it, manipulate it or enrich it in any way [18]—without doing what ([9], p. 18) calls “mental gymnastics”. A few complications are important here:

First, affordances are agent-relative (or, interchangeably for our purposes, co-constituted by the agent and the environment). When we say that the ambient energy array carries information about affordances, we should be read as saying that it does so when we keep a certain agent fixed, or that it does so as parameterized by a concrete agent.

Second, there is some debate in the literature about whether the presence or absence of affordances should be nomologically necessitated by the ambient energy array [21]. That is to say, whether the probability of the presence of a certain affordance given a certain configuration of the ambient energy array should always be 0 or 1—what [18] call *specification*—or just made highly (im)probable by it [9]. In the model we develop in the sequel, we follow Chemero in endorsing this latter probabilistic characterization, which we take to be ecologically more plausible, as it does not require that the ambient energy array be always and everywhere unambiguous. In any event, nomological necessitation is a special case of probabilistic correlation.

Finally, it is common for ecological psychologists to claim that “[t]he idea of ecological information developed by J. J. Gibson has no aspects in common with the idea of information as it is understood by cognitivism” ([17], p. 49), echoing ([15], p. 232). If “information as understood by cognitivism” means information as described in Shannon’s theory of information (see below), this is an exaggeration. If the ambient energy array makes the presence of an affordance more (un)likely, or even necessitates its presence (absence), then, trivially, the mutual information between a random variable, the values of which are possible configurations of the ambient energy array, and another random variable, the values of which are the presence or absence of a certain target affordance, is necessarily nonzero. See Section 4 for the characterization of mutual information.

## 3. Multi-Modal Perception and Synergistic Affordances

There are simple scenarios in which ecological information about some affordance is present in the structured energy of *only one* ambient energy array, pertaining to only one sensory modality. (What counts as a sensory modality is itself a vexed question in this debate. We can assume an ecological-psychology understanding thereof, perhaps along the lines developed in [22].)

For example, a walkable surface can be perceived as such by relying only on the set of regularities in ambient light that can be taken in visually. For the purposes of this paper, we can grant that, in these simple cases, perception of affordances results from the direct pickup of ecological information. This can be seen as a stipulation: when there is only one source of affordance-related information, perception counts as direct. We note, in passing, that this is conceding a lot to the defender of direct perception: deep learning [23] teaches us that extracting ecologically relevant features (e.g., the presence of food, or of stairs) from a single source (e.g., an array of pixels) is a computationally complex process, far from direct under any reasonable definition of “direct”. See [24].

In any case, ecological information about affordances is often the result of complex interactions between several ambient energy arrays, targeted by several different sensory modalities, in a multi-dimensional space, that do not meet this definition of “direct”. One way to develop this idea is Stoffregen and Bardy’s notion of a *global array* [18]. The main idea is that, in the general case, the value of an affordance can be recovered only from ecological information present in all ambient energy arrays considered jointly, but possibly not in subsets thereof. By only considering each of them separately it is not necessarily (and perhaps not typically) possible to pinpoint affordance values to the best available accuracy. We will call these *multimodal affordances*.

Ref. [18] claims that the perception of multimodal affordances in the global array is *also* direct. We do not feel that direct perception has been characterized in a clear enough manner to reach a verdict on this issue. What we propose to do in what follows is to develop a formalization of some of the key notions in the debate, in terms of the so-called *partial information decomposition* framework, so that the trade-offs of taking some act of perception as direct are more sharply in view.

## 4. Information Theory and Lossy Communication

### 4.1. Basic Concepts

As we have seen, the perception of multimodal affordances relies upon the pickup of information present in patterns in the global array. We now introduce tools to quantify to which extent each of the ambient energy arrays that jointly constitute the global array carries affordance-related information, and to which (possibly different) extent the global array does too. We will rely on information theory for this.

Information theory [25] is a mathematical framework that characterizes optimal transmission of information through a typically noisy channel. In this framework, information is a quantity that measures the degree of uncertainty in a random variable. In this work, we treat single ambient energy arrays as random variables that are combined into another random variable—the global array. Thus, multimodal affordance perception is constrained by how these random variables interact with each other. The way information theory formalizes the dependency between two random variables *X* and *Z* is *mutual information*, I(X;Z):

(1) $$\begin{array}{cc} I(X;Z) & =E_{x,z}\left[\log\frac{p(x,z)}{p(x)p(z)}\right] \end{array}$$

(2) $$\begin{array}{cc} \; & =H(X)-H(X|Z) \end{array}$$

where the entropy of a random variable *X*, or H(X), is defined as

(3) $$\begin{array}{c} H\left(X\right)=-E_{p(x)}\left[\log p\left(x\right)\right] \end{array}$$

One way to think of the mutual information between *X* and *Z* is as the reduction in uncertainty (i.e., entropy) of *X* once the value of *Z* is known. Mutual information is symmetric, so it can also be formulated in the other direction; that is, as the reduction in uncertainty about *Z* when *X* is known.

As can be seen, Equation (1) only considers two random variables, which makes it inadequate for our current purposes, where at least three random variables are involved: two (or more) single ambient energy arrays, and the resulting global array.

### 4.2. PID and Synergistic Information

In such higher-dimensional systems, where the information flows from at least two random variables to a third one, we can make use of multivariate mutual information, which, for three random variables, is defined as

(4) $$\begin{array}{c} I(X,Z;Y)=I(Y;X)-I(Y;Z|X) \end{array}$$

One problem with Equation (4) is that it neglects the possibility of information interaction between the set of random variables. It may be, for example, that both *X* and *Z* carry the same pieces of information about *Y* (say, that for some particular ecological situation, what ambient light says about the current landscape of affordances, and what sound waves say about it, is pretty much the same). It may also be that each of *X* and *Z* carries a unique piece of information about *Y*; or that each carries no information about *Y* on their own, but *when put together* they do. Any arbitrary combination of these three possibilities might be the case as well.

Unfortunately, this inquiry goes beyond the scope of classic information theory. The framework of *partial information decomposition* (also PID henceforth, [26]) has been recently formulated as an effort to formalize precisely the ways in which information flows in such multivariate systems. In particular, PID defines three possible interactions between the random variables of a system, informally introduced above, corresponding to three different kinds of information (groups of) variables can carry: redundant, unique, and synergistic. Unique information measures the amount of information that is only present in one random variable, but not the others. Redundant information measures the amount of information available in more than one random variable. Finally, synergistic information measures the amount of information carried by a group of random variables as a whole, but not contained in their individual contributions. Our analyses in this paper rely chiefly on the synergistic components in the PID.

The PID approach is still relatively new, and its formal underpinnings still in flux. Several definitions of synergistic information (and the attendant unique and redundant information notions) have been offered in recent years, all of them with advantages and shortcomings. Among these, we will rely on the mathematical definition of synergistic information provided by [27,28]. Given a set of *n* random variables $X=\{X_{1},X_{2},\ldots ,X_{n}\}$, where n≥2, and a random variable *Y*, they define the synergistic information in **X** about *Y* as follows:

(5) $$\begin{array}{c} I_{\mathrm{syn}}\left(\left\{X_{1},\ldots ,X_{n}\right\};Y\right)=I\left(X_{1\ldots n};Y\right)-I_{union}\left(\left\{X_{1},\ldots ,X_{n}\right\};Y\right) \end{array}$$

where union information is computed as follows:

(6) $$\begin{array}{c} I_{union}\left(\left\{X_{1},\ldots ,X_{n}\right\};Y\right)\equiv \min_{\begin{array}{c} Pr^{*}\left(X_{1},\ldots ,X_{n},Y\right)\\ \mathrm{subject}\;\mathrm{to}:\;Pr^{*}\left(X_{i},Y\right)=Pr\left(X_{i},Y\right)\;\forall i \end{array}}I^{*}\left(X_{1\ldots n};Y\right) \end{array}$$

We can use the Lagrangian method (as we do in a maximum entropy problem) to approximate the optimal distribution in the minimization of the right-hand side [27,28,29]. This definition captures the intuitive idea of synergistic information: the information, $I(X_{1\ldots n};Y)$, that the system as a whole (or joint random variable) $X_{1\ldots n}$ carries about a target variable *Y* is greater than the information, $I_{union}$, that the aggregation of all individual variables, $\left\{X_{1},\ldots ,X_{n}\right\}$, does: the difference, in Equation (5), is the synergistic component. One important reason to rely on this definition of synergy is that it has well-defined bounds. In particular, it is an upper bound on the WholeMinusSum (WMS) synergy [30], which underestimates the synergy in a system, and a lower bound on the $S_{max}$ measure [26], which overestimates it. In addition, Equation (5) exhibits some desirable properties, such as nonnegativity, which early attempts at quantifying interaction information, such as the *interaction information* [31], do not have. (Another recently proposed measure of interactions and dependencies is the so-called *O-information* [32,33]. We will restrict ourselves here to measures in the PID tradition. We would like to thank an anonymous reviewer for pointing us to this alternative body of work).

A common example of a synergistic system is the XOR logic gate, defined by the truth table in Table 1. We can use this simple example to illustrate how synergistic information is not stored in either of the random variables, $X_{1}$ and $X_{2}$, alone but in their combination. First, let us evaluate the information that each input random variable $X_{i}$ carries about the target variable *Y*. Assuming all inputs are uniformly distributed, the mutual information between each input and output is

(7) $$\begin{array}{cc} I(X_{i};Y) & =H\left(X_{i}\right)-H\left(X_{i}|Y\right) \end{array}$$

(8) $$\begin{array}{cc} \; & =H\left(X_{i}\right)-H\left(X_{i}\right)=0 \end{array}$$

Looking closely at Table 1, we see that knowing the value of $X_{i}$ (where i∈{1,2}) does not reduce the initial 1 bit uncertainty of *Y*. For example, knowing that $X_{1}=0$ does not change the initial probabilities p(Y=0) and p(Y=1), which entails $H\left(X_{1}|Y\right)=H\left(X_{1}\right)$. *Mutatis mutandis* for $X_{2}$. Thus, adding the mutual information of the individual components of the XOR gate leads to zero information about the output variable: $I\left(X_{1};Y\right)+I\left(X_{2};Y\right)=0$.

We now evaluate the mutual information between the target variable *Y* when both inputs are considered as a whole $\left\{X_{1},X_{2}\right\}$:

(9) $$\begin{array}{c} I(\left\{X_{1},X_{2}\right\};Y)=1 \end{array}$$

In this case, the uncertainty about the *Y* is completely resolved once both $X_{1}$ and $X_{2}$ are known. Since the information about *Y* is not in any random variable in isolation, but only in their union, information can only flow when the system is considered as a whole, rather than the sum of its parts. This is precisely the intuition behind synergistic information.

### 4.3. Communication

In our model, affordance-related information (e.g., about the presence of food) is conveyed by two energy arrays (e.g., ambient light and sound waves) that causally affect distinct sensory modalities (visual and auditory, in the example). We model the multimodal perception of affordances according to Shannon’s mathematical theory of communication [25]. Roughly speaking, a communication pipeline consists of (a) a source that generates messages; (b) an encoder that sends an encoded signal of the messages through a typically noisy channel; and (c) a decoder that generates faithful estimates of the source messages based on the incoming encoded signals.

(As an aside, we note that Shannon’s communication theory does not require the source messages and the decoder’s estimates to lie in the same dimensional space. For example, we could design a communication pipeline where the source messages are sensory observations at the retinal level and the output of the decoder is an action that depends on visual input. In this scenario, the dimensionality of the source messages is going to be significantly higher than the space of possible actions: $R_{messages}\gg R_{actions}$).

For our specific case of study, we treat each encoder as a sensory modality that receives inputs from a single ambient energy array; the signals can be thought of as neural patterns of activation, perhaps; and the decoder as some cognitive sub-system downstream that generates the affordance percept.

**Figure 1:**
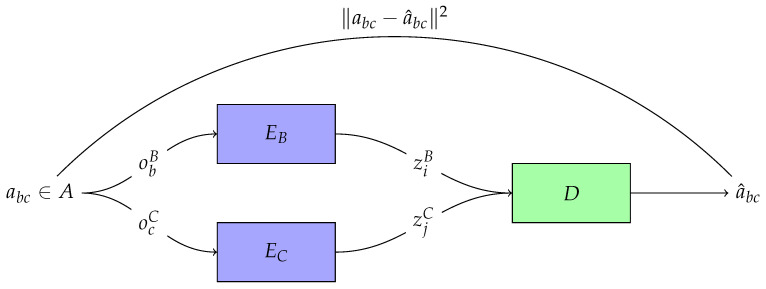
Communication model used to formalize the perception of multimodal affordances. An affordance, $a_{bc}$, is co-instantiated by the energy array states $o_{b}^{B}$ and $o_{c}^{C}$. Then, encoders $E_{B}$ and $E_{C}$ encode each sensory observation as $z_{i}^{B}$ and $z_{j}^{C}$, respectively. Given those signals, the decoder *D* generates an estimate, ${\hat{a}}_{bc}$, of the affordance value $a_{bc}$.

In this multimodal-affordance perception setup, we slightly extend the main Shannonian model by introducing *two* distinct sources (one per energy array) along with their corresponding encoders (one per sensory modality). Each source message is transmitted to its corresponding encoder, which produces a signal. Finally, a single decoder takes incoming pairs of signals from the encoders to generate an affordance estimate (see Figure 1). We can examine the information interaction between the encoded signals and the affordance by using the tools described in Section 4.2.

### 4.4. Lossy Compression

Shannon’s lossless source coding theorem [25] states that any source can be compressed up to its entropy with negligible error. For example, given a discrete random variable *X* that can take four possible states with the following probability distribution p(X)={0.5,0.2,0.2,0.1}, applying Equation (3), we observe that the maximum achievable error-free compression is 1.76 bits. When that is the case, all the information at the source can be perfectly recovered at the end of the communication pipeline by the decoder.

However, cognition operates under limited cognitive resources (due to the cost of metabolic processes, and other biological constraints, [34,35,36,37]), which makes lossless compression, and therefore, lossless communication, rarely achievable. To model such limitations, we impose a capacity constraint: the two modality-specific encoders cannot simply relay all of the information present in their target energy array to the downstream decoder. Formally, this means that the maximum transmission rate *R* (i.e., number of transmitted bits per symbol) achievable by the channel is lower than the entropy of the energy array *O*: R<H(O).

What this means is that the encoder cannot uniquely encode the source messages (i.e., different source messages are mapped onto the same signal). This creates some uncertainty at the decoder, thus making perfect reconstruction of the affordance matrix unfeasible in general. When lossless communication is not viable, a sub-field of information theory called *rate-distortion theory* [38] defines optimal lossy compression. The core idea underlying this theory is that fidelity in communication is governed by the trade-off between transmitted information and the expected distortion level of the source estimates. Formally, this trade-off is captured by the rate-distortion function, which defines the minimum mutual information I(X;Z) (i.e., maximum level of compression) between two random variables *X* and *Z* (source input and its compressed representation, respectively) given some tolerable expected distortion L of the source estimates $\hat{X}$ generated from *Z*. To avoid confusion in our notation, we will use *D* to refer to the decoder (Section 5), and L to refer to the expectation over any arbitrary loss function or distortion measure (e.g., MSE or Hamming distance). The rate-distortion function is ([39], chapter 10):

(10) $$\begin{array}{cc} R(L) & =\min_{q\left(z|x\right):L_{q(x,z)}\leq L}I\left(X;Z\right) \end{array}$$

where *q* is the optimal encoding distribution over *Z* that satisfies the expected distortion constraint and the rate *R* is an upper bound on the mutual information

(11) $$\begin{array}{c} R\geq I(X;Z) \end{array}$$

which follows from the data processing inequality. The measure of distortion L is arbitrary and will depend on the actual task to which the lossily compressed information will be put.

The goal in lossy compression is to minimize the rate *R* without exceeding a given expected distortion L. For our case study of multimodal affordances, each encoder can only send a maximum of *L different* signals such that $R_{L}<H\left(O\right)$. This is, of course, precisely what happens in brains, where the information present, e.g., at the retina, cannot be losslessly reconstructed from the activity of any downstream neural population. Under such constraint, a perfect estimate $\hat{A}$ of the multimodal affordance *A* becomes unachievable; that is, $L(A,\hat{A})>0$. It is now clear why our multimodal perception scenario can be seen as a rate-distortion problem. Even though we are not explicitly computing the rate-distortion function in our experiments, we approximate it algorithmically by minimizing the expected distortion of the affordance estimates given a fixed transmission rate at the encoders (see Section 5.3).

Importantly, while the rate-distortion function is an optimal way to quantify the amount of compression given some distortion constraint, it does not provide any insight into the specific algorithmic implementation to achieve such optimal compression. For this reason, we not only quantify the amount of information transmitted but also examine how these resources are utilized, by calculating the *spatial entropy* of signals (see Section 4.5).

### 4.5. Spatial Entropy

In our model, signals are distributed both probabilistically and spatially. Due to the constraints mentioned above, each encoder has fewer available signals than there are possible energy array states, which forces them to subsume sets of states under single sensory estimates. The spatial distribution of the signals provides insight into which states of the energy array are being represented as which states. To measure this, we use spatial entropy, as characterized in [40], to account for this spatial information:

(12) $$\begin{array}{c} H_{Cl}\left(X\right)=-\sum_{i=1}^{n}d_{i}p\left(x_{i}\right)\log p\left(x_{i}\right) \end{array}$$

Here $d_{i}$ is the average Euclidean distance between signal $x_{i}$ and all other signals. By doing this, we can weight the entropy definition in Equation (3) using the average distance between each sensory signal in the encoding space. Intuitively, for a given distribution over signals, the more spatially spread they are (i.e., the higher *d* is), the higher the spatial entropy. Higher spread among signals suggests that the encoder is giving a fuller picture of the energy array. Conversely, the more densely packed signals in the encoding space are, the fewer spatially distinct aspects of the energy array are being captured.

## 5. Methods

### 5.1. Model Description

This is how we model global arrays: we express an “affordance landscape” as a 2-dimensional, m×n matrix *A*, where each dimension corresponds to one ambient energy array (we will also call these dimensions *basic properties* in what follows). We can think of these dimensions as the model equivalents to, respectively, ambient light and ambient sound, for example. The first dimension (energy array) has *m* possible states; the second one, *n* possible states.

*Sensory observations*, $O^{B}\in R^{m}$ and $O^{C}\in R^{n}$, record the possible values each energy array can take, such that $O^{B}=\left[o_{1}^{B},o_{2}^{B},\ldots ,o_{m}^{B}\right]$ and $O^{C}=\left[o_{1}^{C},o_{2}^{C},\ldots ,o_{n}^{C}\right]$. We define an affordance matrix $A\in R^{m\times n}$ as follows:

(13) $$\begin{array}{cc} A & =\left[\begin{array}{cccc} a_{11} & a_{12} & \ldots & a_{1n}\\ a_{21} & a_{22} & \ldots & a_{2n}\\ \vdots & \vdots & \ddots & \vdots \\ a_{m1} & a_{m2} & \ldots & a_{mn} \end{array}\right] \end{array}$$

where each entry $a_{bc}$ gives the value of the target affordance when the two ambient energy arrays are observed to be in state $o_{b}^{B}$, and $o_{c}^{C}$, respectively.

Modality-specific encoders $E_{B}:o_{b}^{B}\mapsto z_{i}^{B}$ and $E_{C}:o_{c}^{C}\mapsto z_{j}^{C}$ receive these observations, $o_{b}^{B}$ and $o_{c}^{C}$, respectively, and map them to encoded signals, $z_{i}^{B}$ and $z_{j}^{C}$, respectively, that are sent downstream to a decoder $D:\left(z_{i}^{B},z_{j}^{C}\right)\mapsto {\hat{a}}_{bc}$, that generates an estimate ${\hat{a}}_{bc}$ of the current affordance value $a_{bc}$. *A*, *O*, *Z*, and $\hat{A}$ are random variables, while *E* and *D* are functions. The communication pipeline for a 1-dimensional affordance specified by $O^{B}$ is assumed to form the following Markov chain

(14) $$\begin{array}{c} A\overset{f}{\mapsto }O^{B}\overset{E_{B}}{\mapsto }Z^{B}\overset{D}{\mapsto }\hat{A} \end{array}$$

where each component is only conditionally dependent on the previous one. The end goal of the system is to transmit just as much mutual information $I(A;\hat{A})$ as needed to generate faithful enough estimates $\hat{a}$ of the target affordance value *a*.

Encoders are not *directly* causally sensible to the affordance, but only through the basic properties that co-specify the affordance. Whatever we take “direct perception” to imply, it has to be compatible with this fact. Still, the property of interest for the agent is the affordance value: it is with this property that it has to engage in order to generate adaptive behavior. That is to say, the agent’s goal (as ecological psychologists and embodied cognitive scientists rightly point out) is not to reconstruct sensory stimuli (i.e., basic properties), but to minimize their uncertainty about the current value of the affordance.

Once each encoder sends the signals downstream, the decoder’s job is to generate a faithful estimate of the property of interest. We assume that the codebook is shared by the encoder and decoder, so the decoder knows the inverse mapping from encoded signals back to sensory observations and therefore can reconstruct the optimal expected affordance value given that information. To evaluate the “goodness" of those estimates, we use the Mean Squared Error (MSE) between *A* and $\hat{A}$ as a distortion measure L of the generated estimates:

(15) $$\begin{array}{c} L_{MSE}\left(A,\hat{A}\right)=\frac{1}{\left|O\right|}\sum_{bc\in O}{\left(a_{bc}-{\hat{a}}_{bc}\right)}^{2}\;\mathrm{where}\;O=\left[\left(o_{b}^{B},o_{c}^{C}\right)|b\in O^{B},c\in O^{C}\right] \end{array}$$

which computes the squared distance between each estimate and the actual affordance value. We define each decoder’s estimate ${\hat{a}}_{bc}$ as the expected affordance value corresponding to the observations encoded under the same signal:

(16) $$\begin{array}{c} D\left(z_{i}^{B},z_{j}^{C}\right)={\hat{a}}_{bc}=\frac{1}{\left|O\right|}\sum_{bc\in O}a_{bc}\;\mathrm{where}\;O=\left[\left(o_{b}^{B},o_{c}^{C}\right)|b\in E_{B}^{-1}\left(z_{i}^{B}\right),c\in E_{C}^{-1}\left(z_{j}^{C}\right)\right] \end{array}$$

where $z_{i}^{B}$ and $z_{j}^{C}$ are the *i*th and *j*th signals encoding observations $o_{b}^{B}$ and $o_{c}^{C}$, respectively, via the mappings $E_{B}\left(o_{b}^{B}\right)$ and $E_{C}\left(o_{c}^{C}\right)$. The above expression estimates each affordance value by taking the expectation over all affordance values that correspond to each pair of observations encoded in each modality. We use *O* to refer to the set of pairs of the Cartesian product between the observations obtained through the inverse mapping of the encoders. As a crucial part of this work is to understand whether the perception of multimodal affordances entails any intermediate processing of the energy arrays, we also measure whether the whole system is keeping track of sensory observations. In particular, we compute the sensory estimates that the decoder can generate via the encoder’s inverse mapping:

(17) $$\begin{array}{c} {\hat{o}}_{b}^{B}=\frac{1}{\left|O\right|}\sum_{o\in O}o\;\mathrm{where}\;O=\left[o_{b}^{B}|b\in E_{B}^{-1}\left(z_{i}^{B}\right)\right] \end{array}$$

where, similarly as before, $z_{i}^{B}\in Z^{B}$ is the *i*th signal that encodes the sensory observation $o_{b}^{B}$. This expression computes each sensory estimate by averaging over all observations $O^{B}$ that are mapped onto the same signal $z_{i}^{B}$.

### 5.2. Encoding Strategies

We investigate two different encoding strategies. First, we evaluate the *direct encoding* strategy, which tries to maximize information about the property of interest (i.e., the affordance value). In this strategy, each encoder generates a mapping such that the content of the signals directly maximizes affordance information. Since each encoder is only sensitive to one dimension of the affordance matrix, the best they can do is to transmit as much information about the expected affordance value of the dimension they are causally sensitive to. Formally, the expected affordance value corresponding to dimension *B* (and, *mutatis mutandis*, *C*) can be defined as

(18) $$\begin{array}{c} A^{B}=E_{c}\left[a_{bc}\right]\;\forall b\in O^{B} \end{array}$$

Given this, the *direct encoding* strategy can be formalized as follows:

(19) $$\begin{array}{c} \underset{Z^{B}}{arg max}I\left(A^{B};{\hat{A}}^{B}\right) \end{array}$$

In particular, each one-dimensional affordance estimate can be obtained by

(20) $$\begin{array}{c} {\hat{A}}_{b}^{B}=D\left(E_{B}\left(o_{b}^{B}\right)\right)=\frac{1}{\left|O\right|}\sum_{o\in O}E_{c}\left[a_{oc}\right]\;\mathrm{where}\;O=\left[o_{b}^{B}|b\in E_{B}^{-1}\left(z_{i}^{B}\right)\right] \end{array}$$

We intend for this strategy to be a formalization of the direct perception claim that affordance-related information can be simply picked up from the energy array. Our two direct encoders do just that: simply pick up as much affordance-related information from their proprietary arrays as they can. For our toy example, we directly compute $I(A^{B};{\hat{A}}^{B})$. However, we observe that in a more complex scenario, the spatial distribution of the signals is key to determining the *usefulness* of the encoding strategy (see Section 4.5), which we address below. Thus, to provide a simple measure for Equation (19), we approximate this quantity through $L_{MSE}\left(A^{B},{\hat{A}}^{B}\right)$ as follows:

(21) $$\begin{array}{cc} I(A^{B};{\hat{A}}^{B}) & =H\left(A^{B}\right)-H\left(A^{B}|{\hat{A}}^{B}\right) \end{array}$$

(22) $$\begin{array}{cc} \; & =H\left(A^{B}\right)+E_{p}\left[p\left(a^{B}|{\hat{a}}^{B}\right)\right] \end{array}$$

(23) $$\begin{array}{cc} \; & \geq H\left(A^{B}\right)+E_{p}\left[q\left(a^{B}|{\hat{a}}^{B}\right)\right] \end{array}$$

(24) $$\begin{array}{cc} \; & \approx -L_{MSE}\left(A^{B},{\hat{A}}^{B}\right) \end{array}$$

where we choose a Gaussian distribution *q* as an approximation to the true distribution *p*. As $H(A^{B})$ is a constant (i.e., the affordance matrix does not change), maximizing mutual information amounts to minimizing the mean-squared error.

In contrast to direct encoding, we examine an *indirect encoding* strategy that merely aims at supplying the decoder with the signals that will allow *the decoder* to come up with the best possible reconstruction of affordance value. In this strategy, encoders do not make any assumptions as to whether this requires them to squeeze as much affordance-related information as possible or not. The main question to analyze is how much *sensory* information signals carry when encoders follow this strategy. In particular, we want to understand to what extent information in the signals depends on

(25) $$\begin{array}{c} \underset{Z^{B}}{arg max}I\left(O^{B};{\hat{O}}^{B}\right) \end{array}$$

which would imply that indirect encoders end up prioritizing the transmission of information about sensory data. If that is the case, then the perception of affordance-related information would be mediated by the integration of the sensory signals of each modality, and therefore, indirect. Similarly to ${\hat{A}}^{B}$, each sensory estimate in ${\hat{O}}^{B}$ can be computed using Equation (17). We approximate Equation (25) using the mean-squared error, as performed before, and the spatial entropy. The justification for using the mean-squared error is equivalent to the one provided before. Regarding the spatial entropy, we use it to examine how the spatial distribution of signals contributes to minimizing $L_{MSE}\left(O^{B},{\hat{O}}^{B}\right)$. As mutual information is symmetric, we follow the other direction to obtain the entropy of the sensory estimates:

(26) $$\begin{array}{cc} I(O^{B};{\hat{O}}^{B}) & =H\left({\hat{O}}^{B}\right)-\underset{=0}{\underset{︸}{H({\hat{O}}^{B}|O^{B})}} \end{array}$$

(27) $$\begin{array}{cc} \; & =H({\hat{O}}^{B}) \end{array}$$

where the last term in the right-hand side of Equation (26) arises from using a deterministic encoder. Then, we simply replace $H({\hat{O}}^{B})$ by its spatial entropy counterpart $H_{Cl}\left({\hat{O}}^{B}\right)$ defined in Section 4.5. Using spatial entropy can provide a deeper understanding of how the spatial distribution of signals contributes to achieving (near) optimal encoding strategies, beyond just considering the probability distribution of signals.

For the sake of simplicity, throughout the whole model description and further experiments, we assume that (i) all random variables are discrete; (ii) both $O^{B}$ and $O^{C}$ are uniformly distributed; and, (iii) the distribution of the other random variables (*O*, $\hat{O}$, *Z*, *A*, $\hat{A}$) is given by the frequency of its values.

### 5.3. Encoder Optimization

In our experiments, we run a simple optimization algorithm to approximate optimal encoder strategies. Suppose we have two encoders, each of which has a repertoire of *n* possible signals. The pseudocode for this optimization is given in Algorithm 1. As for the “relevant MSE” in line 14 of Algorithm 1: in the direct perception scenario we use MSE_direct: each encoder is individually optimized to minimize their MSE; while in the indirect case, we use MSE_indirect: we find the pair of encoders that *jointly* minimize it.
**Algorithm 1** Encoder Optimization  1:b← dimension of $O^{B}$ energy array  2:c← dimension of $O^{C}$ energy array  3:m← number of signals available for the $E^{B}$ encoder  4:n← number of signals available for the $E^{C}$ encoder  5:A←b×c matrix                                                                                                                                                ▹ affordance landscape  6:$A_{b}\leftarrow$ a vector with the means of A rows                                                        ▹ affordance landscape as seen by the $E^{B}$ encoder  7:$A_{c}\leftarrow$ a vector with the means of A columns                                                  ▹ affordance landscape as seen by the $E^{C}$ encoder  8:RUNS ← how many different random starting points  9:LENGTHOFRUN ← how many optimization steps10:**for** RUNS times **do**11:    $ENC_{1}\leftarrow$ random vector of integers from 1 to *m*, of size *b*12:    $ENC_{2}\leftarrow$ random vector of integers from 1 to *n*, of size *c*                               ▹ Random initialization of the two encoders13:    **for** LENGTHOFRUN times **do**14:        Compute the relevant MSEs (see explanation in main text).15:        For each encoder: randomly modify the signal to which one particular (also random) observation is mapped. If the resulting MSE is lower than the one calculated above, keep the new encoder; otherwise, discard it.16:    **end for**17:**end for**18:Keep the encoders with the lowest MSE  19:**function** MSE_direct(encoder)20:    decoder ← all zeros vector with size <number of signals available at the encoder>21:    $\hat{A}\leftarrow$ all zeros vector with size <length of encoder (i.e., number of observations)>22:    MSE s← all zeros vector with size <length of encoder (i.e., number of observations)>23:    **for** i← 1 to number of signals available at the encoder **do**24:        decoder[i]← the mean of all observations (from 1 to length of encoder) that the encoder maps onto signal *i*25:    **end for**26:    **for** i← 1 to length of encoder **do**27:        $\hat{A}\left[i\right]\leftarrow$ decoder[encoder[i]]                                                                                ▹ what the decoder produces given the signal28:        $MSEs\left[i\right]\leftarrow {\left(A\left[i\right]-\hat{A}\left[i\right]\right)}^{2}$29:    **end for**30:    Return the mean of MSEs31:**end function**  32:**function** MSE_indirect(encoder1, encoder2)33:    $\hat{A}\leftarrow$ all zeros matrix with dimensions equal to affordance map *A*34:    decoder ← all zeros matrix with dimensions <m×n>                                         ▹ the decoded value given a pair of signals35:    MSEs← an all zeros matrix with dimensions equal to affordance map *A*36:    **for** i← 1 to *m* **do**37:        **for** j← 1 to *n* **do**38:           decoder[i,j]← the mean of all observations that the encoders maps onto signals *i* and *j* respectively39:        **end for**40:    **end for**41:    **for** i← 1 to *b* **do**42:        **for** j← 1 to *c* **do**43:           $\hat{A}\left[i\right]\leftarrow$ decoder[encoder1[i],encoder2[j]]                                                       ▹ what the decoder produces given the signals44:           $MSEs\left[i\right]\leftarrow {\left(A\left[i\right]-\hat{A}\left[i\right]\right)}^{2}$45:        **end for**46:    **end for**47:    Return the mean of MSEs48:**end function**

While there is no guarantee that this algorithm will find the optimal encoders, first, in our tests it consistently lands on encoders that are optimal or close to optimal; and, second, it is the same procedure for all tests so results for different strategies are (barring some unexpected bias) fully comparable.

It is not always easy to reconstruct an algorithm from this kind of pseudocode. The fully explicit code is available on the following Github repository: https://github.com/MigueldeLlanza/SynergisticPerception (accessed on 3 May 2024).

### 5.4. Information-Theoretic Measures

We rely on the BROJA measure from the *dit* python package [41] to compute the synergistic measure defined in Equation (5). Similarly, we adapt the code from the *Spatentropy* R package [42] to measure the spatial entropy measure defined by Equation (12).

### 5.5. Data

We first evaluate the direct-perception claim with a toy example using a synthetic 4×4 affordance matrix that exhibits synergistic properties. This simple scenario is useful to examine in detail how information is processed in each encoding strategy. Then, we further investigate the direct perception claim using realistic images from the CIFAR-100 dataset [43]. We chose the “people” superclass of CIFAR-100 as the data source due to its simplicity compared to other classes. When solving Equation (5), each unique RGB pixel value in the range [0,255] is treated as a different value of the random variable *A*. For this reason, calculating the synergy becomes computationally intractable. To overcome these computational demands we transform each image to grayscale and reduce the number of unique pixel values to 5 using K-means clustering. Here we assume the following tradeoff: calculating the synergy becomes tractable at the expense of reducing the image quality. The goal in this second scenario is to explore information processing in a context with plausible sensory inputs (visual in this case). To make an artificial multimodal setup, we consider each dimension of an image as a different energy array that causally affects each encoder independently. That is to say, we interpret each image as a 2-dimensional affordance matrix, where each pixel value (i.e., affordance value) is assumed to be co-defined by the instantiation of each energy array. For example, the top-right pixel value of an m×n image is co-defined by the first value of the first energy array (i.e., row 0) and the last value of the second energy array (i.e., column *n*).

## 6. Results

### 6.1. Toy Example

In this section, we first analyze a toy model of a cognitively bounded agent whose goal is to perceive a multimodal affordance. In this setup, the maximum achievable rate is less than the entropy of the receptor fields, so the encoders cannot account for all the variability in the input, which makes it a rate-distortion problem. In addition, each encoder is only sensitive to one dimension of the affordance matrix, corresponding to the one basic property it is causally sensitive to. Following the previous description, for a $A\in R^{m\times n}$, and a set of observations $O^{B}$, the dimensionality of the encoded signals will be $Z^{B}\in R^{m}$:

(28) $$\begin{array}{c} Z^{B}=\left[E_{B}\left(o_{1}^{B}\right),E_{B}\left(o_{2}^{B}\right),\ldots ,E_{B}\left(o_{m}^{B}\right)\right] \end{array}$$

where the set of signals for a specific energy array ($O^{B}$ in this case) is a vector of encoded observations. If we think of the energy array $O^{B}$ as color, then an instantiation of that random variable $o_{1}^{B}$ could be read as *color red* (i.e., B=color and 1=red). As the constrained encoder cannot send a different signal per color, some colors will be subsumed under the same signal following a *many-to-one* mapping. In this scenario, the decoder has to deal with some uncertainty about what sensory observations caused the received encoded signals, so we assume that the decoding process relies on the expected affordance value corresponding to all the observations mapped onto the same signal.

Finally on to our toy example. Assume the following 4×4 affordance matrix *A*

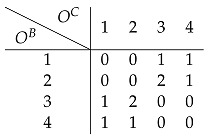
(29)
that depends on two energy arrays $O^{B}=\left[1,2,3,4\right]$ and $O^{C}=\left[1,2,3,4\right]$ that we can think of as, e.g., color and loudness. Assuming a channel capacity of 1 bit, each encoder can only send two signals (0 and 1). As mentioned before, each encoder is sensitive to the expected affordance value per dimension: $A_{b}^{B}=E_{c}\left[a_{bc}\right]$ and $A_{c}^{C}=E_{b}\left[a_{bc}\right]$, respectively. For example, the expected affordance values corresponding to dimension *B* are:

(30) $$\begin{array}{cc} A_{1}^{B} & =E_{c}\left[a_{1c}\right]=\frac{1}{4}\left[0+0+1+1\right]=0.5 \end{array}$$

(31) $$\begin{array}{cc} A_{2}^{B} & =\frac{1}{4}\left[0+0+2+1\right]=0.75 \end{array}$$

(32) $$\begin{array}{cc} A_{3}^{B} & =\frac{1}{4}\left[1+2+0+0\right]=0.75 \end{array}$$

(33) $$\begin{array}{cc} A_{4}^{B} & =\frac{1}{4}\left[1+1+0+0\right]=0.5 \end{array}$$

Each encoder alone could potentially discriminate two different expected affordance values, 0.5 and 0.75. Similarly, each encoder is only able to discriminate between two different energy array states (i.e., two different colors or two different sound levels), as it can transmit 1 bit of information.

#### 6.1.1. Direct Encoding

Under this strategy, each encoder sends signals that maximize affordance information. In this example, $E_{B}$ and $E_{C}$ generate the following mappings:

(34) $$\begin{array}{cc} E_{B}\left(o_{b}^{B}\right) & =\left\{\begin{array}{cc} 0, & \mathrm{if}\;o_{b}^{B}\in \left\{1,4\right\}\\ 1, & \mathrm{if}\;o_{b}^{B}\in \left\{2,3\right\} \end{array}\right\} \end{array}$$

(35) $$\begin{array}{cc} E_{C}\left(o_{c}^{C}\right) & =\left\{\begin{array}{cc} 0, & \mathrm{if}\;o_{c}^{C}\in \left\{1,4\right\}\\ 1, & \mathrm{if}\;o_{c}^{C}\in \left\{2,3\right\} \end{array}\right\} \end{array}$$

For example, if the affordance value $a_{13}$ is the case, then $o_{1}^{B}$ (e.g., red color) and $o_{3}^{C}$ (e.g., loud sound), and the encoded signals will be $Z^{B}=0$ and $Z^{C}=1$. Here, each encoder is trying to maximize affordance information given the receptive field it is sensitive to. For instance, subsuming energy array states 2 and 3 under the same signal can be understood as attributing high affordance value to those states, and low affordance value to the pair of values 1 and 4. This is an intuitive strategy to follow, as each encoder is trying to provide as much relevant information as possible on its own.

As shown before, given a pair of signals, the best the decoder can do is to apply Equation (16) to compute the expectation of the affordance value corresponding to the sensory observations mapped onto those signals. In the current example, the decoded expected affordance ${\hat{a}}_{13}$ is:

(36) $$\begin{array}{cc} D(Z^{B}=0,Z^{C}=1) & =\frac{1}{4}\left[a_{12}+a_{13}+a_{42}+a_{43}\right] \end{array}$$

(37) $$\begin{array}{cc} \; & =\frac{1}{4}\left[0+1+1+0\right]=\frac{2}{4}=0.5 \end{array}$$

Following the same procedure for all affordance values and corresponding sensory observations, we end up with the following estimate $\hat{A}$ of the affordance matrix:

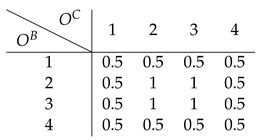
(38)
whose expected distortion can be evaluated by computing Equation (15):

(39) $$\begin{array}{c} L_{MSE}\left(A,\hat{A}\right)=0.44 \end{array}$$

Here, the strategy of the encoders is to maximize affordance information as each signal maximizes the expected affordance value along its basic property dimension. In particular, the expected affordance value is higher when the basic property value is either 2 or 3, and lower when it is 1 or 4. Computing Equation (17) for all possible $O^{B}$, we have the following expected decoder’s receptive field estimate:

(40) $$\begin{array}{ccccc} O^{B} & 1 & 2 & 3 & 4\\ Z^{B} & 0 & 1 & 1 & 0\\ {\hat{O}}^{B} & 2.5 & 2.5 & 2.5 & 2.5 \end{array}$$

so when $O^{B}\in \left\{2,3\right\}$, it entails a high affordance value and the opposite when $O^{B}\in \left\{1,4\right\}$. The same holds for $O^{C}$ (as the affordance matrix in this toy example is symmetric, all the results shown for the energy array *B* hold for *C*). Interestingly, maximizing affordance information is at odds with conveying information about the basic property. All sensory information is destroyed by this encoding strategy since the decoder collapses all possible sensory states into the same estimate 2.5; that is, no matter what signals are sent downstream, the best the decoder can do is to map them onto the same value, thus destroying all the information in the receptive fields. This type of encoder is the one we call *direct*, as it does not at all keep track of the sensory stimuli it is sensitive to:

(41) $$\begin{array}{cc} I(O^{B};{\hat{O}}^{B}) & =H\left(O^{B}\right)-H\left(O^{B}|{\hat{O}}^{B}\right) \end{array}$$

(42) $$\begin{array}{cc} \; & =H\left(O^{B}\right)-H\left(O^{B}\right)=0 \end{array}$$

but, instead, tries to capture as much information as possible about the property of interest *A*:

(43) $$\begin{array}{ccccc} A^{B} & 0.5 & 0.75 & 0.75 & 0.5\\ {\hat{A}}^{B} & 0.5 & 0.75 & 0.75 & 0.5 \end{array}$$

leading to $I(A^{B};{\hat{A}}^{B})=1$. (Note that $p\left(O^{B},{\hat{O}}^{B}\right)=p\left(O^{B}\right)$ because the encoder is deterministic: $p\left(O^{B},{\hat{O}}^{B}\right)=p\left({\hat{O}}^{B}|O^{B}\right)p\left(O^{B}\right)=p\left(O^{B}\right)$).

#### 6.1.2. Indirect Encoding

Can we do better with the same resources? The answer is yes. We now examine whether Equation (25) is needed to capture the synergistic interactions in the system. In this example, a synergistic strategy is achieved by the following mappings:

(44) $$\begin{array}{cc} E_{B}\left(o_{b}^{B}\right) & =\left\{\begin{array}{cc} 0, & \mathrm{if}\;o_{b}^{B}\in \left\{1,2\right\}\\ 1, & \mathrm{if}\;o_{b}^{B}\in \left\{3,4\right\} \end{array}\right\} \end{array}$$

(45) $$\begin{array}{cc} E_{C}\left(o_{c}^{C}\right) & =\left\{\begin{array}{cc} 0, & \mathrm{if}\;o_{c}^{C}\in \left\{1,2\right\}\\ 1, & \mathrm{if}\;o_{c}^{C}\in \left\{3,4\right\} \end{array}\right\} \end{array}$$

**Figure 2:**
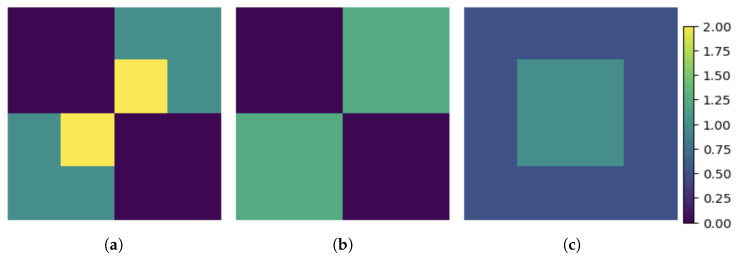
Affordance estimates of the toy model. (**a**) Affordance matrix. (**b**) Indirect estimate. (**c**) Direct estimate.

Following the same steps as in the direct encoding, the expected affordance estimate is (see Figure 2, which shows the raw affordance matrix along with the corresponding direct and synergistic estimates)

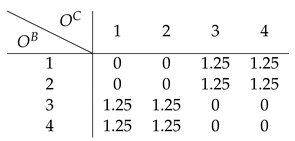
(46)
which leads to a better-expected distortion compared to the direct strategy:

(47) $$\begin{array}{c} L_{MSE}\left(A,\hat{A}\right)=0.09 \end{array}$$

How much receptive field information is transmitted in this scenario? Again, using Equation (17) the decoder’s estimate of the receptive field inputs given the received encoded signals is:

(48) $$\begin{array}{ccccc} O^{B} & 1 & 2 & 3 & 4\\ Z^{B} & 0 & 0 & 1 & 1\\ {\hat{O}}^{B} & 1.5 & 1.5 & 3.5 & 3.5 \end{array}$$

As can be seen, *all* the information about the sensory states that can be captured with a 1-bit encoder is preserved

(49) $$\begin{array}{cc} I(O^{B};{\hat{O}}^{B}) & =H\left(O^{B}\right)-H\left(O^{B}|{\hat{O}}^{B}\right) \end{array}$$

(50) $$\begin{array}{cc} \; & =2-1=1 \end{array}$$

as there is 1 bit of information transmitted through the whole communication pipeline. In particular, the decoder’s receptive field estimate is 1.5 when $O^{B}\in \left\{1,2\right\}$, and 3.5 otherwise. In this scenario, the encoded signals can be interpreted as carrying information about the receptive fields rather than directly about the affordance value. Importantly, this strategy leads to an efficient use of the available resources, as the system transmits at its maximum capacity, which is a 1 bit rate (i.e., sending either a 0 or 1, which is then translated by the decoder as 1.5 or 3.5). Symmetrically, no affordance information is stored in any of the encoders alone:

(51) $$\begin{array}{ccccc} A^{B} & 0.5 & 0.75 & 0.75 & 0.5\\ {\hat{A}}^{B} & 0.625 & 0.625 & 0.625 & 0.625 \end{array}$$

as $I(A^{B};{\hat{A}}^{B})=0$. This is why indirect encoding works better: as the information of the affordance value is carried synergistically by the two energy arrays, it pays off to relay an estimate of those very arrays so that the downstream decoder can then reconstruct these synergistic components. If each encoder tries to maximize affordance-related information directly, “going it alone”, the synergistic components will not be transmitted, and the decoder will not be able to exploit them. Table 2 summarizes the results shown for each strategy in the toy example.

(Note that one could swap entries $a_{42}$ and $a_{23}$ of Equation (29) to create an affordance matrix with synergistic information, where both strategies would result in the same affordance estimate).

This toy model is, of course, constructed precisely to show clearly what we want it to show. In the next section we make the same point, but now relying on statistically natural stimuli.acces

### 6.2. CIFAR-100

**Figure 3:**
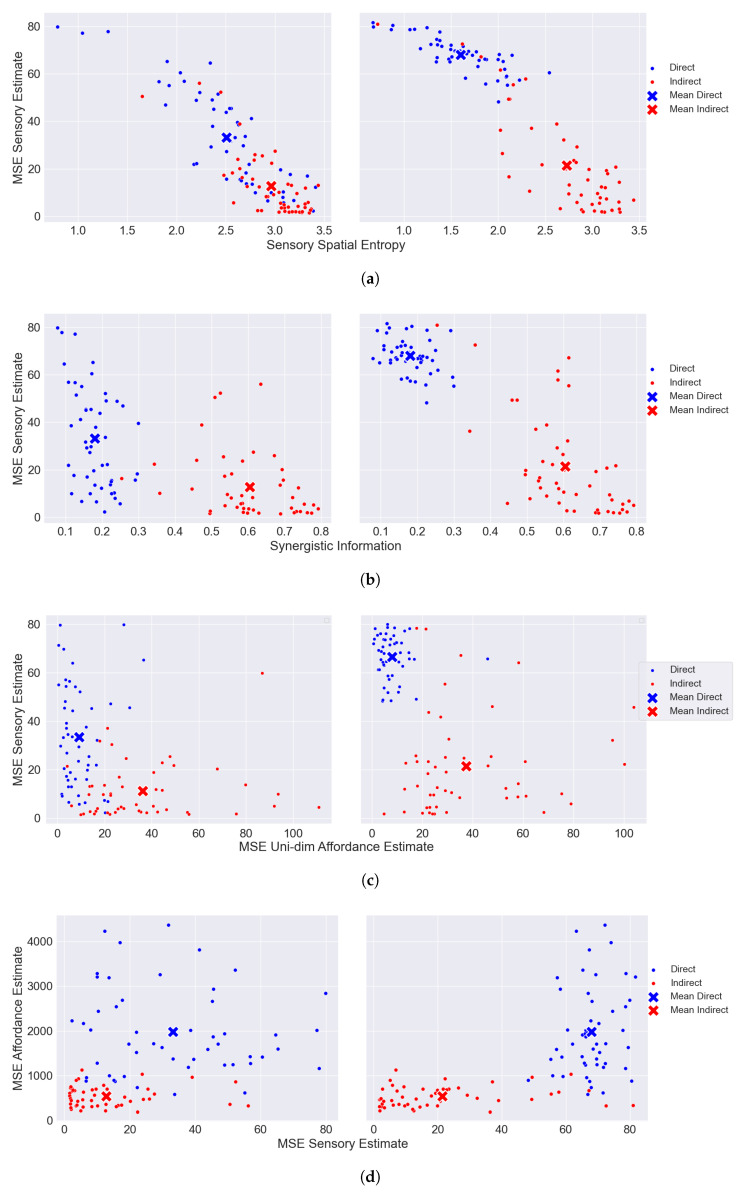

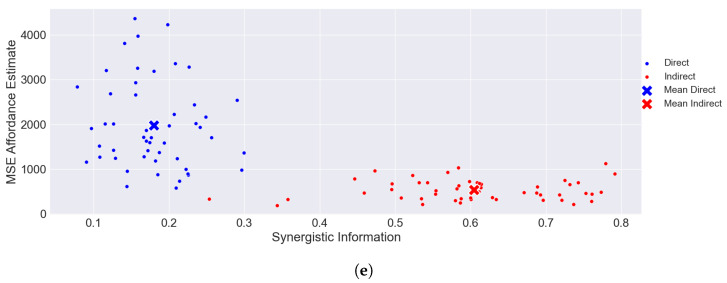
Results for different metrics for both the direct and the indirect encoding strategies when the capacity constraint is set to 8 signals per encoder; that is, each encoder can, at most, encode 8 out of 32 dimensions of the input. In (**a**–**d**), the left plot corresponds to the results obtained for encoder *B*, while the right plots correspond to the results for encoder *C*. In each plot, we show the results per data point (i.e., CIFAR-100 images) and the mean corresponds to the point of the means of each dimension. (**a**) Sensory accuracy as a function of spatial entropy. (**b**) Sensory accuracy as a function of synergistic information. (**c**) Sensory accuracy as a function of uni-dimensional affordance accuracy. (**d**) Affordance accuracy as a function of sensory accuracy. (**e**) Affordance accuracy as a function of synergistic information.

After showing the behavior of each encoding strategy in a toy model, we now show the results using CIFAR-100 data as affordance landscapes. To evaluate the direct perception of synergistic affordances, we examine how sensory information is related to affordance information under each encoding–decoding strategy (i.e., direct and indirect). In Figure 3, we show the results for the case in which the maximum capacity is constrained to 3 bits per encoder. (We stick to 3 bits due to the computational costs of solving Equation 5). That is, each encoder can only encode 8 dimensions (using $2^{3}$ signals) out of the 32 possible they are causally sensitive to (CIFAR-100 images have a 32×32 dimension). In particular, each energy array is defined as $O^{B}=\left[0,1,\ldots ,31\right]$ (sensible to the image rows; i.e., horizontal information) and $O^{C}=\left[0,1,\ldots ,31\right]$ (sensible to the image columns, i.e., vertical information).

We show the following results grouped by strategy for each encoder dimension: (i) Figure 3a shows the correlation between sensory estimates (“MSE sensory estimates”) and sensory spatial entropy; (ii) Figure 3b shows the correlation between sensory estimates and synergistic information; (iii) Figure 3c shows the correlation between sensory estimates and estimates of each dimension of the affordance; (iv) Figure 3d illustrates how affordance estimates (“MSE Affordance Estimate”) are correlated with sensory estimates; and (v) Figure 3e shows the correlation between affordance estimates and synergistic information.

The main source of evidence supporting the claim that the direct perception of multimodal synergistic affordances is suboptimal can be found in Figure 3e. There it is shown how minimizing affordance distortion is achieved by maximizing the synergistic information (i.e., $I_{syn}\left(\left\{Z^{B},Z^{C}\right\};A\right)$) present in the affordance matrix, thus supporting the claim that *synergy makes direct perception inefficient*. In the same line, Figure 3d shows how indirect encoders (red dots) manage to significantly minimize the expected distortion of the affordance value by minimizing the expected distortion of the sensory observations. This suggests that, at least in some contexts, *a near-optimal encoding strategy has to keep track of sensory observations to improve the estimates of the property of interest.*

What kind of information does each encoding strategy aim to maximize? Figure 3c shows a trade-off between sensory and affordance information: maximizing one quantity (Equation (21)) is at the expense of minimizing the other (Equation (17)), in line with Section 5.2. Encoders following the direct strategy seem to individually maximize affordance information to the detriment of discarding sensory information, while the ones following the indirect strategy behave oppositely.

**Figure 4:**
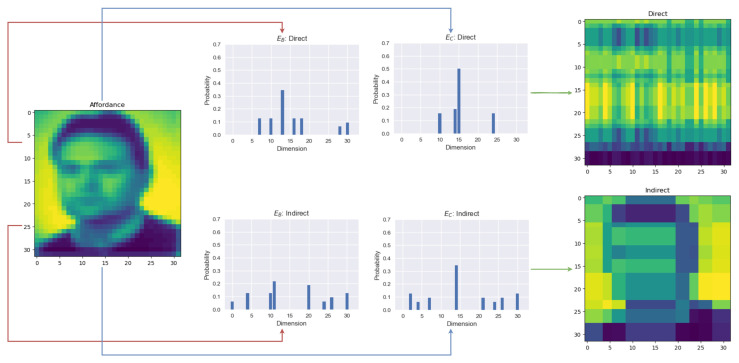
Encoded signals of a CIFAR-100 image used as an affordance landscape (left) and the resulting estimates (right) per each encoding–decoding strategy: direct (top) and indirect (bottom). As can be seen, the indirect encoded signals are more spread out across their possible states (32 dimensions) and have higher entropy (i.e., closer to a uniform distribution) than the direct encoding. Thus, indirect encodings exhibit a higher spatial entropy.

Figure 3a shows how encoders that minimize the sensory distortion maximize their spatial entropy to account for as much variability about the sensory observations as possible. Thus, examining the spatial distribution of signals is necessary to account for the encoding behavior. All this is consistent with the efficient coding claim that neurons are tuned to the statistical properties of their sensory input by maximizing their information capacity (i.e., entropy) [44,45], which in this case is captured by their spatial entropy. As can be seen, in Figure 4, indirect encoders create a more spread encoding of the signals compared to the direct strategy. Note that the strategy found by the algorithm can sometimes have some degree of redundancy. This happens when information conveyed by more than one signal is collapsed onto the same dimension of the sensory observation. In the direct strategy shown in Figure 4, $I(O;\hat{O})<3$, since less than 8 dimensions of the sensory dimensions are being captured. Therefore, spatial entropy sheds some light on how the encoders have to map the inputs onto signals to convey the relevant information downstream.

Next, we explore whether the relation between sensory and affordance distortion is related to the synergistic nature of the affordance matrix. In Figure 3b, we see how the synergistic information is tightly related to sensory distortion. In particular, indirect encoders capture sensory information by increasing the synergistic information they carry about the affordance matrix, compared to the direct ones.

Note that in Figure 3a–d, the difference between each strategy is greater between encoders *C* (figures on the right). This is mainly due to the structure of the data. Encoders *C* are sensible to the vertical dimension of CIFAR-100 data, which is more likely to contain most of its pixel variability in fewer dimensions. For instance, an image of a standing person has its main vertical variance along the pixel columns where the person is standing. However, the horizontal dimension of that same image contains variability in a wider range of pixel rows. A direct strategy will use most of its information capacity to capture high-density regions of affordance-related information, at the expense of missing sensory-related information, which leads to an encoding that is highly penalized in synergistic contexts.

In addition, we also computed the *p*-values to evaluate the statistical significance of the results shown in each of the subplots in Figure 3. For example, we computed the p-value to evaluate the statistical significance of the synergistic strategy over the direct one regarding the “MSE Sensory Estimate” results. For all measures, the results of the indirect encoding–decoding pair were statistically significant compared to the direct behavior (p≪0.05).

These results suggest that the perception of synergistic multimodal affordances heavily relies on keeping track of sensory information, which is needed to capture as much synergistic information as possible. Direct strategies cannot capture synergistic interactions because most of the sensory information is destroyed by the encoders, leading to inefficiency. Thus, optimal multimodal perception of synergistic affordances cannot be direct; it requires a modicum of computation to properly combine different streams of information.

## 7. Discussion

### 7.1. Direct Perception and Synergistic Information in Nature

In this work, we have shown how direct perception of synergistic multimodal affordances results in an inefficient pickup of affordance-related information. One could retort that, even if somewhat inefficient, direct perception might still, as a matter of fact, be the prevalent perceptual mechanism underlying adaptive behavior and that, therefore, perception is not mediated by any computational process. While we agree that direct perception might be all there is in certain contexts, there is wide evidence of synergistic multimodal affordances in nature and cognition. For example, [46] provides some evidence that woodboring insects synergistically integrate multimodal cues during host selection. They suggest that these insects synergistically combine both visual and olfactory cues when making host-selection decisions. Another example of multimodal perception can be found in [47]. In their research, they study how rats categorize the orientation of grids (horizontal or vertical) when they rely on either visual, tactile, or visual-tactile information. They show that visual-tactile information is synergistically combined, which results in better performance when categorizing the orientation of the grids. According to our model and results presented above, to properly perceive these synergistic multimodal cues, some degree of inner processing or computation is needed: at least to that extent, perception is indirect.

### 7.2. Direct Perception and the Global Array

What about the possibility, rehearsed above, of directly perceiving the global array in its entirety? We have shown how the global array contains synergistic information that depends on energy arrays that have to be combined through some computations. Could there be a mechanism that allows the direct perception of the global array, without relying on energy-array specific information? At least in some important cases, neurophysiology prevents this—sensory surfaces are quite simply not in physical contact. This is all we are assuming in our model. For one prominent example, the organ of Corti connects to the cortex via the auditory nerve; and the retina connects to the cortex via the optical nerve. Any informational combination of these two sensory inputs has to happen *after* information is relayed through those two, plausibly not fully lossless, nerves. Of course, there is ample evidence that brains integrate information from different sensory modalities in order to guide behavior [48,49,50,51]; and, as an anonymous reviewer has reminded us, this combination can happen as soon as V1 (e.g., [52]). This suggests that cognitive systems generate a single percept by combining incoming signals from each modality in some downstream region [53]. This combination of, first, lossy transmission of sensory information and, then, downstream combination of this information, is what we aim at capturing with our model.

### 7.3. Real Multimodal Data to Study Information Interaction

In this study, we have not used real multimodal data, but interpreted CIFAR-100 images “multimodally”, by considering vertical and horizontal informations independently. For subsequent work, we expect to run similar models on naturalistic, *bona-fide* multimodal data.

## Figures and Tables

**Table 1:** Truth table of an XOR gate.

$X_{1}$	$X_{2}$	*Y*
0	0	0
0	1	1
1	0	1
1	1	0

**Table 2:** Results of the two encoding strategies for affordance reconstruction, synergistic information, sensory state information, and uni-dimensional affordance information.

Strategy	$L_{MSE}\left(A,\hat{A}\right)$	$I_{syn}\left(\left\{Z^{B},Z^{C}\right\};A\right)$	$I(O^{B};{\hat{O}}^{B})$	$I(O^{C};{\hat{O}}^{C})$	$I(A^{B};{\hat{A}}^{B})$	$I(A^{C};{\hat{A}}^{C})$
Direct	0.44	0.25	0	0	1	1
Indirect	0.09	1	1	1	0	0

## Data Availability

All of the code necessary to reproduce the figures and analyses in this paper can be found at https://github.com/MigueldeLlanza/SynergisticPerception (accessed on 3 May 2024).
